# Natural Products and Their Derivatives as Inhibitors of the DNA Repair Enzyme Tyrosyl-DNA Phosphodiesterase 1

**DOI:** 10.3390/ijms24065781

**Published:** 2023-03-17

**Authors:** Alexandra L. Zakharenko, Olga A. Luzina, Arina A. Chepanova, Nadezhda S. Dyrkheeva, Nariman F. Salakhutdinov, Olga I. Lavrik

**Affiliations:** 1 Novosibirsk Institute of Chemical Biology and Fundamental Medicine, Siberian Branch of the Russian Academy of Sciences, 8, Akademika Lavrentieva Ave., Novosibirsk 630090, Russia; 2 N. N. Vorozhtsov Novosibirsk Institute of Organic Chemistry, Siberian Branch of the Russian Academy of Sciences, 9, Akademika Lavrentieva Ave., Novosibirsk 630090, Russia

**Keywords:** natural compound, derivative, TDP1 inhibitors, anticancer therapy, TOP1 inhibitors

## Abstract

Tyrosyl-DNA phosphodiesterase 1 (TDP1) is an important repair enzyme that removes various covalent adducts from the 3′ end of DNA. Particularly, covalent complexes of topoisomerase 1 (TOP1) with DNA stabilized by DNA damage or by various chemical agents are an examples of such adducts. Anticancer drugs such as the TOP1 poisons topotecan and irinotecan are responsible for the stabilization of these complexes. TDP1 neutralizes the effect of these anticancer drugs, eliminating the DNA adducts. Therefore, the inhibition of TDP1 can sensitize tumor cells to the action of TOP1 poisons. This review contains information about methods for determining the TDP1 activity, as well as describing the inhibitors of these enzyme derivatives of natural biologically active substances, such as aminoglycosides, nucleosides, polyphenolic compounds, and terpenoids. Data on the efficiency of combined inhibition of TOP1 and TDP1 in vitro and in vivo are presented.

## 1. Introduction

Tyrosyl-DNA phosphodiesterase 1 (TDP1) is a repair enzyme for “stalled” DNA -topoisomerase 1 (TOP1) cleavage complexes and other 3′ end DNA lesions. TDP1 plays a crucial role in the repair of DNA lesions formed by antitumor drugs such as the TOP1 inhibitors camptothecin, topotecan (a TOP1 poison in clinical use), and irinotecan; therefore, TDP1 is a promising target for adjunctive cancer treatment.

Because homologs of TDP1 have been present in organisms throughout most of evolution [1], there is a real possibility that natural biomolecules could have evolved to interact with this protein as well. In addition, a combination of antitumor and tumor-chemosensitizing properties of drugs can lead to an enhanced therapeutic effect, and many natural compounds have intrinsic antitumor properties. For instance, since the 1980s, approximately 40% of all approved anticancer drugs worldwide have been either natural substances or compounds derived from pharmacophores of natural substances [2]. Natural substances and compounds derived from natural pharmacophores are known to effectively disrupt many critical processes involved in the pathogenesis of many cancer types [3]. 

Therefore, special attention is given to the search for inhibitors of TDP1 among natural compounds and their derivatives. This review provides general information on the structure and function of TDP1, assays of the activity of this enzyme, and compounds of natural or semisynthetic origin (chemically derived from natural compounds) that inhibit TDP1.

## 2. Tyrosyl-DNA Phosphodiesterase 1: General Information

TDP1 was discovered in the yeast *Saccharomyces cerevisiae* as an enzyme that hydrolyzes the covalent bond between a tyrosine residue and a 3′-phosphate group of DNA. Because the only known enzyme that forms the 3′-phosphotyrosyl linkage in vivo is DNA topoisomerase I (TOP1), some authors proposed that the detected activity is involved in the repair of lesions in DNA resulting from the formation of irreversible Top1–DNA complexes [4]. Later, it was shown [5] that some predicted but unidentified genes of eukaryotic organisms contain a sequence that plays a role in the hydrolysis of the covalent bond between a tyrosine residue and a 3′-phosphate group of DNA. This led to the hypothesis that TDP1 or its homologs are present in all eukaryotic organisms that had been studied [5]. 

**Figure 1:**
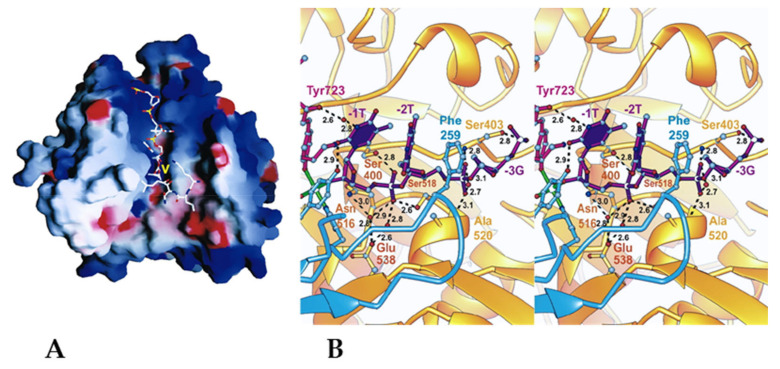
Crystal structure of the complex of TDP1 with the vanadate oxoanion VO_4_^3−^ [6]. (**A**) Red depicts a negative partial charge on the surface, blue depicts positive partial charge. The yellow V indicates the position of the vanadate residue in the active site. The DNA moiety extends above the active site, bound in the narrow, positively charged half of the substrate-binding groove. The peptide moiety is located below the active site, in a relatively neutral part of the wider substrate-binding cleft (Protein Data Bank (PDB) ID: 1NOP). (**B**) Detailed view of contacts between the substrate and amino acid residues in the catalytic site of TDP1. H-bonds between DNA and amino acid residues of TDP1 are indicated.

A crystallographic study has revealed that human TDP1 is a monomer and consists of two topologically similar α-β-α domains, and each domain carries a conserved amino acid sequence, so-called HKN motif characteristic of TDP1 orthologs (for human TDP1: H263 K265 N283 and H493 K495 N516) [6,7]. These two HKN motifs are brought together in a tertiary structure, thereby forming a catalytic site located inside a narrow asymmetric substrate-binding channel, which has a positive charge for binding single-stranded DNA (Figure 1A) [6,7].

To study the catalytic center of TDP1, it has been crystallized with the vanadate oxoanion VO_4_^3−^ mimicking a phosphate group attached to the catalytic tyrosine residue of TOP1, with a 6-mer oligonucleotide, and a peptide having a sequence corresponding to amino acid residues 720–727 of TOP1. Both HKN motifs stabilized the vanadate by means of six hydrogen bonds (H-bonds) between nitrogen atoms of HKN residues and oxygen atoms of the vanadate (Figure 1B) [6,7]. Single-stranded DNA was stabilized by a network of polar interactions involving (i) 5′-phosphate groups of the three nucleotides adjacent to the phosphotyrosyl bond (T-1, T-2, and G-3, Figure 1B) and (ii) amino acid residues S400, S403, K469, S518, K519, and A520. DNA stabilization is implemented via both hydrophobic and polar interactions, and therefore TDP1 is able to recognize a substrate regardless of its nucleotide sequence. 

This observation is consistent with the fact that TOP1–DNA complexes can form on different sequences, and TDP1 should perform the catalysis efficiently regardless of the nucleotide composition of this sequence [6,8].

**Figure 2:**
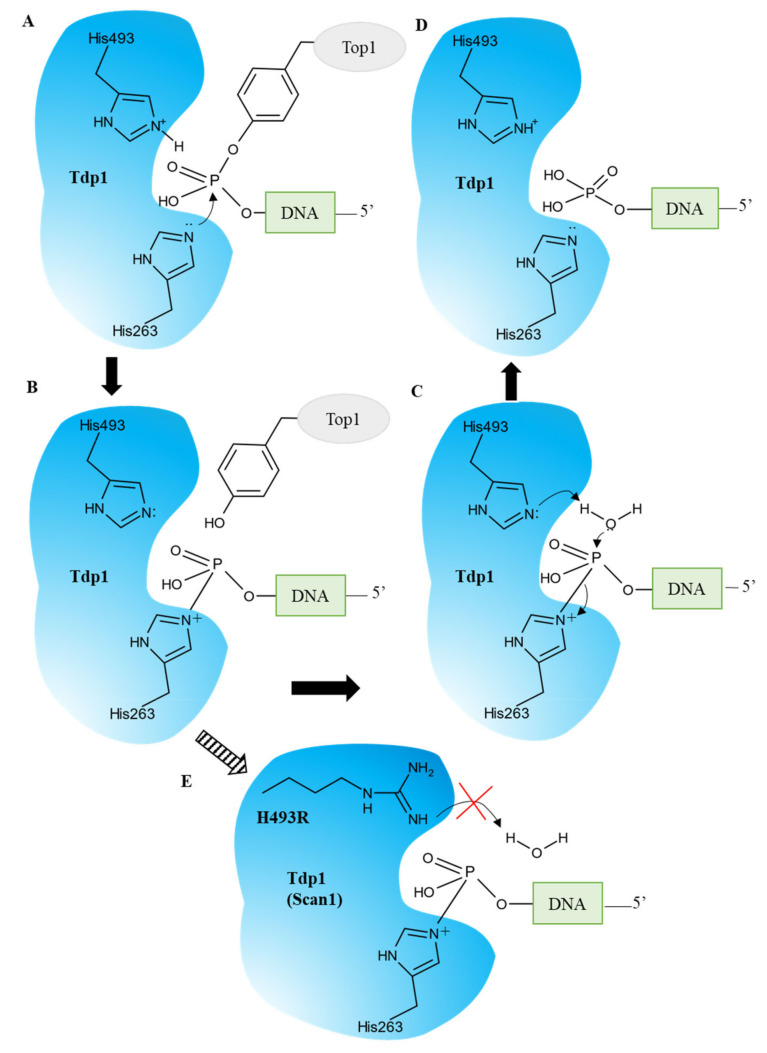
TDP1′s catalytic cycle. (**A**) The nucleophilic attack of the phosphodiester backbone by the imidazole N2 atom of H263. H493 donates a proton to the tyrosyl moiety of the leaving group. (**B**) The phosphohistidine covalent intermediate. (**C**) The second nucleophilic attack mediated by a water molecule activated by H493. (**D**) The emergence of the final 3′-phosphate product and free TDP1. (**E**) The SCAN1 mutation (H493R) leads to the accumulation of the TDP1–DNA intermediate and a fall in the TDP1 turnover rate.

TDP1 can carry out the catalytic process independently of cofactors or metals. TDP1 hydrolyzes the TOP1–DNA complex in two steps with the formation of a transient covalent complex [5] (Figure 2). The first step is a nucleophilic attack of the phosphotyrosyl bond of TOP1–DNA by His263 provided by the N-terminal HKN motif. Residue His493 from the opposite HKN motif acts as an acid and donates a proton to TOP1s leaving the tyrosine group (Figure 2A). A transient covalent phosphamide bond arises between His263 and the 3′ end of the DNA (Figure 2B). A nucleophilic attack by a water molecule activated by His493 hydrolyzes the phosphohistidine intermediate and free TDP1 (Figure 2C) [7]. The outcome is DNA with a free 3′-phosphate end (Figure 2D) which needs subsequent processing by polynucleotide kinase phosphatase (PNKP) [9,10].

A natural mutation, H493R, in the TDP1 gene leads to a neurodegenerative disease called spinocerebellar ataxia syndrome with axonal neuropathy (SCAN1), and the cause of this disease is not a loss of function or activity of TDP1 but a gain of function: the formation of stable covalent complexes of the mutant enzyme with DNA (Figure 2E) [11,12]. It has been hypothesized that inhibition of the mutant enzyme for preventing the formation of these complexes can improve the clinical condition of patients and/or slow down the development of the disease.

### 2.1. TDP1 as a Target for Anticancer Therapy

Originally, TDP1 was discovered as an enzyme capable of hydrolyzing the 3′-phosphotyrosyl bond between TOP1 and a 3′-end of DNA [4]. This enzyme has been extensively studied, and it has been found that, aside from the hydrolysis of the 3′-phosphotyrosyl bond of TOP1, TDP1 can cleave a wide range of physiological and pharmacological 3′-blocking lesions [11] and has the ability to cleave 5′-phosphotyrosyl bonds [13,14].

The use of TDP1 as a target of anticancer therapy for enhancing the action of TOP1 inhibitors was proposed by Nash’s research group [4] as early as 1996. Later, it was shown that overexpression of TDP1 is associated with chromosomal instability [15] and is observed in such types of cancer as non-small-cell lung cancer [16] and colorectal cancer [17] as well as in cell lines derived from breast cancer [18] and some rhabdomyosarcomas [19]. Additionally, overexpression of TDP1 protects cells from both camptothecin and its derivatives aimed at suppressing TOP1 activity [20] and from etoposide, which is intended to suppress the activity of TOP2 [20], which forms 5′-phosphotyrosyl bonds. Human cell lines with a *TDP1* gene mutation that reduces enzymatic activity and *TDP1* knockout mice are hypersensitive to camptothecin [21,22,23,24]. Conversely, during elevated expression of TDP1, camptothecin or its derivatives cause fewer DNA lesions [20,25,26,27,28].

It has also been demonstrated that suppression of TDP1 activity causes hypersensitivity of cells not only to camptothecin and its derivatives but also to other DNA-damaging agents [11]. For instance, inhibition of TDP1 activity enhances the sensitivity of cells to anticancer drugs temozolomide [29] and bleomycin as well as to hydrogen peroxide, ionizing radiation, and methyl methanesulfonate [13]. In glioblastoma cells resistant to temozolomide-based chemotherapy, TDP1 depletion induces noticeable sensitization to this drug [29]. 

All of the above indicates that the suppression of the activity of TDP1 can increase the cytotoxicity of various antitumor drugs aimed at damaging tumor DNA and can help in the fight against drug-resistant tumors. A possible therapeutic benefit of the combined use of such substances and TDP1 inhibitors is stronger suppression of cancer cell growth and/or a reduction in the dose of conventional chemotherapy.

It was shown in [24,30] that a *TDP1* gene knockout in vertebrate systems did not affect the survival of the organism; accordingly, it can be theorized that a decrease in the activity of TDP1 will be tolerated by patients fairly well during chemotherapy. Furthermore, SCAN1 patients have spinocerebellar atrophy (causing ataxia that debuts in the second decade of life) but show no increase in cancer predisposition, myocardial toxicity, immunodeficiency, or other problems related to impaired DNA repair [1]. Furthermore, as we show below, most TDP1 inhibitors do not manifest acute toxicity or well-pronounced cytotoxicity; these observations also allow us to count on the good tolerability of adjunctive treatment with these drugs.

### 2.2. Approaches to the Search for Inhibitors and Methods for Determining the Activity of the Inhibitors

One of the approaches in the search for TDP1 inhibitors is a virtual screening of known natural metabolites and their derivatives. A popular model for virtual docking of inhibitors is the PDB structure of a quaternary complex, 1NOP (Figure 1) [6], TDP1 crystallized in the presence of a tyrosine-containing peptide, a single-stranded DNA oligonucleotide, and vanadate. This complex mimics the transition state in the first step of the catalytic reaction. Researchers also employ PDB structure 1MU7: a human TDP1–tungstate complex [7]. Tungstate is an analog of phosphate in the transition state of the enzyme’s active site. Among the studied potential inhibitors of TDP1, compounds having an intrinsic antitumor activity, preferably inhibitors of TOP1, receive special attention. The combination of inhibitory properties toward both enzymes within a single molecule is attractive because TDP1 inhibitors have been proposed as adjunctive drugs for antitumor therapy based on TOP1 poisons. The traditional method of TDP1 activity testing is the electrophoretic separation of the substrate from the product of phosphotyrosine cleavage from the 3′ end of an oligonucleotide [7]. This method is extremely low-throughput and does not permit an accurate assessment of the kinetic parameters of the reaction catalyzed by TDP1, although this technique is still used for semiquantitative assessment of the effects of various compounds on the activity of this enzyme.

For fast screening of libraries of compounds, investigators have developed several colorimetric and fluorescent assays of TDP1 activity, such as, for example, a fluorescence-based assay involving oligonucleotide and nucleotide substrates containing 3′-(4-methylumbelliferone)-phosphate [31]. All these assays are based on the ability of TDP1 to cleave off various 3′ end DNA adducts, including dyes [8,31,32], and to cleave a phosphodiester bond in molecules other than DNA [33,34]. These methods are simple and inexpensive, the activity of the enzyme either increases absorption at a certain wavelength [31,33] or induces fluorescence [34], or a fluorophore/quencher pair is separated, where fluorescence intensity also strengthens [32,35,36]. Several exotic, complicated, and expensive high-throughput methods of screening for inhibitors of TDP1 should also be mentioned:

1. The electrochemiluminescent (ECL) assay based on ruthenium labels (BV-TAG™) designed to emit light upon stimulation [37].
2. AlphaScreen technology [38], which is based on the separation of a donor/acceptor pair located at different ends of an oligonucleotide. Biotin at the 5′ end of the oligonucleotide is attached to the donor through a complex with streptavidin. The donor emits singlet oxygen when irradiated with light at 680 nm. FITC (fluorescein isothiocyanate) residue at the 3′ end of the oligonucleotide in complex with an anti-FITC antibody is the acceptor. The latter, when colliding with singlet oxygen, emits light of 580–620 nm wavelength. An intact substrate, when mixed with the donor streptavidin and anti-FITC antibody (acceptor), gives a strong AlphaScreen signal, whereas cleavage of the substrate by TDP1 weakens the signal.
3. Gyrasol assay technology [39]. This technology is based on a small-molecule nonfluorescent trivalent metal ion sensor designed to bind to the phosphate backbone of a DNA oligonucleotide. There is a fluorophore at the 3′ end of the oligonucleotide. The fluorescence is suppressed by electron transfer quenching. When the distance to the fluorophore increases, the distance becomes too long for the quenching, and the TDP1 activity can be monitored as an increase in fluorescence intensity.

## 3. Types of Natural Compounds and Their Derivatives as Inhibitors of TDP1 

### 3.1. Aminoglycoside Antibiotics and Nucleosides

**Figure 3:**
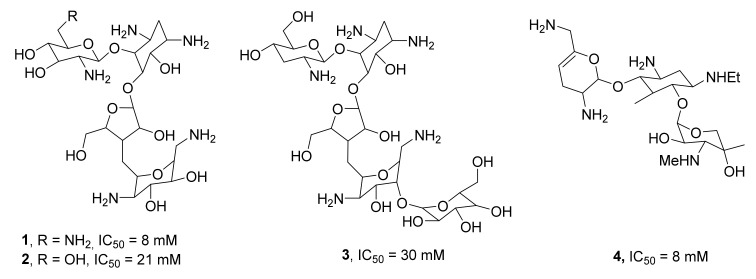
Aminoglycosides as inhibitors of TDP1.

Some of the first discovered pharmacological inhibitors of TDP1 were natural compounds produced by various microorganisms: aminoglycoside antibiotics. It had been previously shown that these antibiotics are noncompetitive inhibitors of ribosomes and suppress the activity of phospholipase D family (PLD) [40,41]. Given that TDP1 belongs to the PLD family [5,6], these antibiotics have been tested for their ability to inhibit TDP1. It has been demonstrated that such antibiotics as aminoglycoside, aminocyclitol, tetracycline, puromycin, and thiostrepton can inhibit both ribosomes and TDP1 [42]. Examples of aminoglycosides are neomycin **1**, paromomycin **2**, lividomycin **3**, and netilmicin **4** (Figure 3). Neomycin has been found to inhibit the interaction of TDP1 with single-stranded and double-stranded substrates, but the inhibition is slightly stronger in the case of a DNA duplex. On the other hand, aclarubicin (an anthracycline drug for treating cancer) can inhibit only the reaction with a double-stranded substrate [42]. Aminoglycoside concentrations that are active toward TDP1 are very high (>5 mM); besides, all aminoglycosides are poorly absorbed in the intestinal lumen and are ineffective when administered systemically.

**Figure 4:**
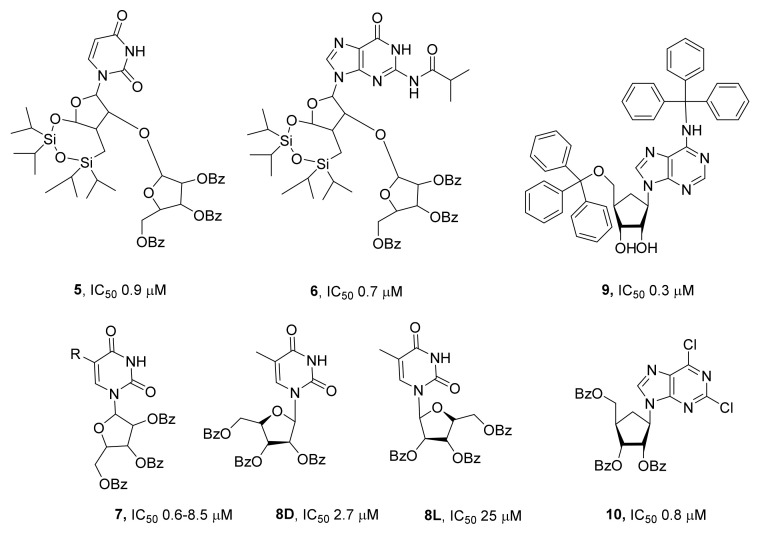
Nucleosides as inhibitors of TDP1.

The research on nucleosides as inhibitors of TDP1 began with disaccharide derivatives [43]. The advantage of disaccharide nucleosides is that they can easily penetrate both the plasma membrane and nuclear membrane of the cell via a nucleoside transporter system similar to the system of transport of various antiviral drugs (acyclovir, zidovudine, and others) and anticancer drugs (cytarabine, cladribine, and gemcitabine) based on nucleosides [44]. The authors of [43] have revealed that disaccharide nucleosides’ half-maximal inhibitory concentrations (IC_50_) toward TDP1 are in micromolar and submicromolar ranges, with tribenzoylated derivatives **5** and **6** having the strongest inhibitory effect: IC_50_ values of 0.9 and 0.7 μM, respectively (Figure 4). These compounds have shown low intrinsic cytotoxicity and a significant synergistic effect when combined with topotecan against immortalized cell line A549 (non-small-cell lung cancer) or WI-38 (lung cells of noncancerous origin).

Additionally, effective inhibitors of TDP1 have been found in a series of monosaccharide nucleoside derivatives **7** (Figure 4) [45]. Lipophilic pyrimidine nucleosides **7** were also reported to have IC_50_ values in low micromolar and submicromolar ranges. The effectiveness of inhibition of TDP1 by these compounds depended on the number of benzoic acid residues attached to the ribose residue: trisubstituted derivatives turned out to be more effective than di- and monosubstituted derivatives. Nucleosides without lipophilic groups showed no inhibitory activity at concentrations up to 50 μM. Compounds of this class were shown to have low cytotoxicity, and some derivatives enhanced the effect of topotecan on HeLa cells. 

The configuration of chiral atoms in the sugar residue of lipophilic nucleosides **7** influences the inhibition of TDP1 [46]. D-lipophilic nucleoside derivatives were shown to inhibit the enzyme more efficiently than L-analogs; for example, the IC_50_ of 1-(2,3,5-tri-*O*-benzoyl-β-D-ribofuranosyl)thymine **8D** was an order of magnitude lower than the IC_50_ for L-ribofuranose isomer **8L** (Figure 4; 2.7 and 25 μM, respectively).

In [47], derivatives of purine and pyrimidine nucleosides containing benzoic acid residues on the ribose ring were synthesized as well as nucleosides having trityl substituents both in the ribose and in the nitrogenous base (Figure 4; compound **9**). The obtained compounds turned out to be effective TDP1 inhibitors (IC_50_ values in the range 0.3–7.0 μM) and in vitro sensitizers of the action of topotecan against the HeLa cell line. This sensitizing effect was independent of the ability of these compounds to inhibit TDP1. Presumably, this finding indicates the presence of other targets for these compounds in the cell. In that study, it was also demonstrated that this compound class could enhance DNA damage induced by topotecan in vitro (in a comet assay involving a human cell line, HeLa) and potentiates the antitumor effect of topotecan in vivo in a mouse ascitic Krebs-2 carcinoma model. It should be noted that trityl derivative **9** (just as benzoic acid derivative **10**; Figure 4) could sensitize cultured cells to topotecan, aiding their survival, and has little effect on topotecan-induced DNA damage accumulation (compound **10** significantly increases the damage), but in contrast to compound **10**, did not sensitize Krebs-2 carcinoma to topotecan in vivo.

### 3.2. Phenolic Compounds

Phenolic compounds are widespread in nature; they contain a common structural component (an aromatic ring with a hydroxyl substituent), but are very structurally diverse (flavonoids, quinones, stilbenoids, meroterpenoids, and others).

**Figure 5:**
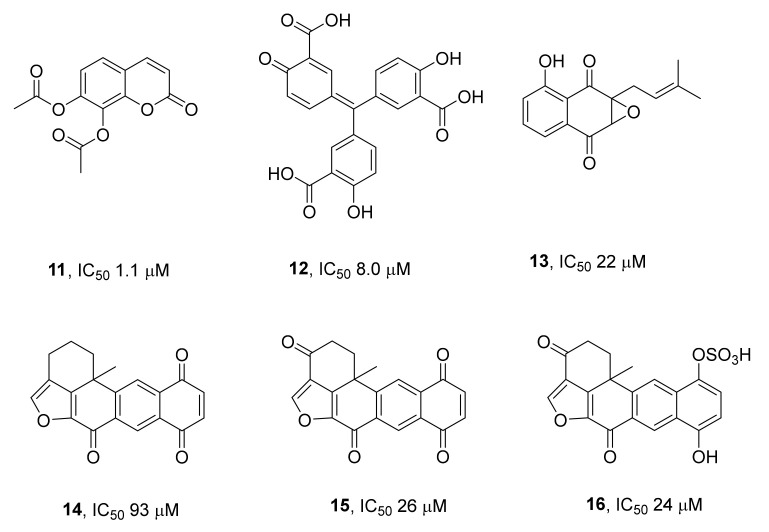
Natural phenolic compounds that inhibit TDP1.

One of the first articles in this field was an exploratory study [48], where the authors aimed to identify inhibitors of TDP1 in extracts of natural substances. They used a test system designed primarily for the search for phenolic compounds. Several new TDP1 inhibitors were identified (Figure 5) that inhibit the activity of this enzyme in micromolar concentrations; most of them were phenolic compounds. Among these, the lowest IC_50_ values were shown by daphnetin diacetate **11** (1.1 µM) and aurintricarboxylic acid **12** (8 µM). Somewhat less active toward TDP1 (IC_50_ = 22–93 µM) were compounds with a quinoid structure: 2,3-epoxy-8-hydroxy-lapachol **13**, xestoquinone **14**, halenaquinone **15**, and halenaquinone sulfate **16**, isolated from the sponge *Xestospongia* sp.

**Figure 6:**
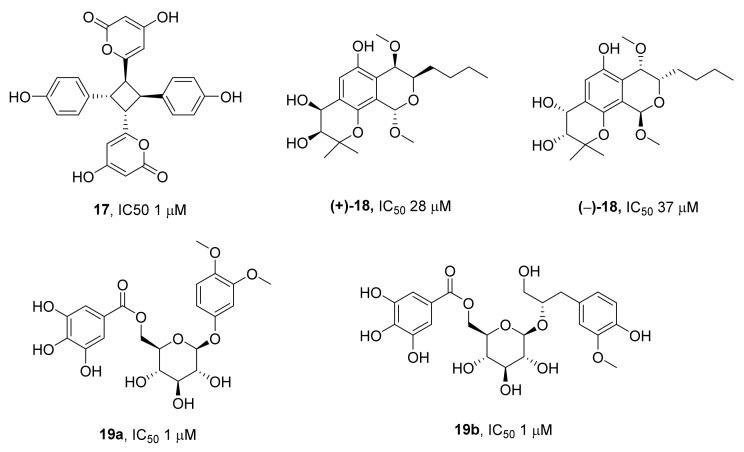
Natural polyphenols as TDP1 inhibitors.

Later, some other secondary metabolites of the polyphenol class of fungal origin were identified as TDP1 inhibitors. It was reported that the secondary metabolite (Achirodimer F, compound **17**) of a teleomorphic fungus from the *Cortinariaceae* family has a noticeable ability to inhibit TDP1 with a 1 μM IC_50_; the compound was isolated [49] from a dichloromethane–methanol extract (Figure 6). Two benzodipyran metabolites featuring a rare pyrano[4,3-h]chromene scaffold have been isolated from the soft-coral-derived fungus *Aspergillus* sp. [50]. In a TDP1 inhibition assay, a racemic mixture of (±)-aspergiletals **18** (Figure 6) showed significantly stronger inhibitory activity toward TDP1 than the (+)- and (−)-enantiomers alone, with IC_50_ values of 6.5, 28, and 37 μM, respectively. The authors concluded that such an increase in the activity seen in the mixture of isomers as compared to individual substances could be a consequence of the synergistic effect. Analogs of macropterantol phenylpropanoid glycoside that were isolated from the bark of the Australian plant *Macropteranthes leichhardtii* (compounds **19a** and **19b**, Figure 6) [51] were also found to be TDP1 inhibitors, with an IC_50_ ~1.0 μM.

Note that both natural phenols and polyphenols inhibit TDP1 at fairly low concentrations. On the basis of these data, a number of researchers have focused on expanding the list of phenolic compounds by chemical modification of natural compounds or by the synthesis of their analogs.

**Figure 7:**
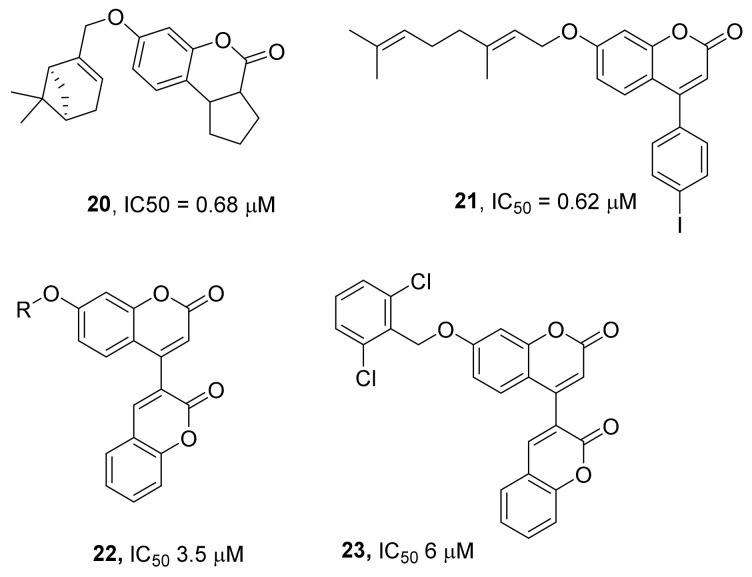
Coumarin derivatives as inhibitors of TDP1.

For instance, continuing the topic of screening of coumarins for the TDP1 inhibitory activity (see daphnetin diacetate **11**, Figure 5) and using the results of a virtual screening of the InterBioScreen library of natural compounds, the authors of ref. [52] have synthesized a series of 7-hydroxycoumarin derivatives containing aromatic and/or monoterpene substituents and evaluated the ability of these compounds to inhibit TDP1. Among terpene-substituted 7-hydroxycoumarins with a saturated cycle annelated on the pyran ring, a derivative with a pinene substituent was found to be the most effective TDP1 inhibitor, with an IC_50_ value of 0.68 μM (compound **20**, Figure 7). This compound has low cytotoxicity (CC_50_ > 100 μM), and its application at nontoxic doses in combination with camptothecin significantly increased the toxicity of camptothecin toward human breast adenocarcinoma MCF-7 cells.

It has been predicted by molecular modeling that the addition of an aromatic substituent at the fourth position of coumarin could improve its binding to the enzyme [53]. Nonetheless, most monoterpene–arylcoumarin hybrids showed similar inhibitory activities against TDP1 (~0.5 μM) and weak cytotoxicity across the entire range of tested concentrations (up to 100 μM). Among arylcoumarin–nopol hybrids and myrtenol–geraniol hybrids, a geranyl-containing derivative (compound **21**, Figure 7) was selected for a study in an animal model. This compound caused a significant increase in the antitumor effect of topotecan on a Krebs-2 model ascitic tumor in vivo, both in terms of the average ascites weight and the number of tumor cells in the ascites. In addition, the combination of topotecan and compound **21** significantly extended the life expectancy of mice with Lewis lung carcinoma as compared to both a control group and groups of mice receiving each monotherapy.

In ref. [54], researchers performed virtual screening of compounds (from the InterBioScreen collection of natural compounds) regarding effects on a model natural TDP1 mutant called SCAN1 (TDP1 protein structure PDB 1MU714 was edited to introduce the histidine-to-arginine (H493R) mutation). Seventeen compounds were identified as inhibitors of SCAN1 and were tested on the purified SCAN1 protein in vitro. Six compounds showed an ability to inhibit SCAN1 with IC_50_ values in the range from 3.5 to 25 μM. The strongest inhibitor of SCAN1 was dicoumarin **22** (IC_50_ = 3.5 μM), followed by its close structural analog **23** with an IC_50_ of 6.0 μM. All the above compounds were also tested for activity against wild-type TDP1, and all six inhibitors proved to be effective; for example, ligand **22** inhibited wild-type TDP1 with an IC_50_ of 99 nM.

**Figure 8:**
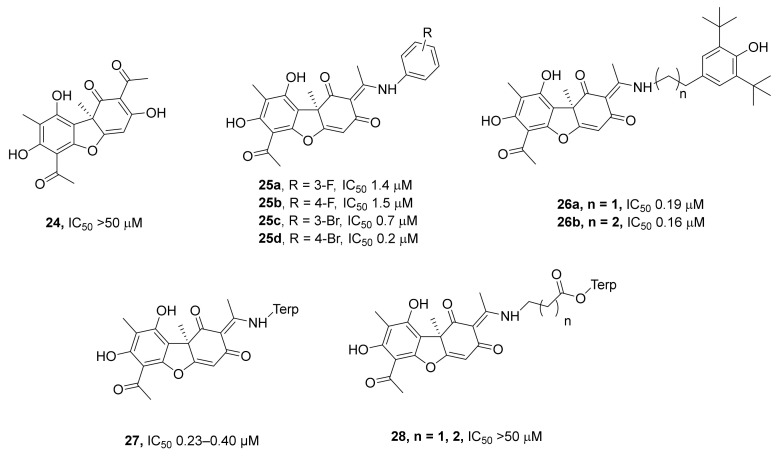
Enamine derivatives of usnic acid as inhibitors of TDP1.

A large series of studies have been devoted to the synthesis and assessment of the TDP1 inhibitory ability of derivatives of a natural compound called usnic acid. This acid (**24**, Figure 8) is a widely accessible secondary metabolite of lichens and belongs to the class of benzofurans [55]. It is amenable to directed chemical transformations via modification of phenolic (A) or triketone (C) rings, resulting in the variation of biological properties among these derivatives [55,56]. It has been found that native usnic acid does not inhibit TDP1, but its derivatives, such as compounds of type **25** (Figure 8), were highly effective [57]. In vitro experiments—regarding the ability of usnic acid derivatives to sensitize the cytotoxic effect of topotecan on cultured cells—showed that in the series of compounds **25**, compounds containing a halogen at the *meta*-position of the aromatic ring are more promising. For instance, the enhancement of camptothecin cytotoxicity (ratio of CC_50_ of pure camptothecin to CC_50_ in the presence of usnic acid enamine) for compound **25a** (R = *m*-F) was found to be 12.8, and for its analog featuring an F atom at the *para*-position (compound **25b**), this enhancement factor was 2.5. The difference was even more pronounced for bromoderivatives **25c** and **25d**: 12.5 for *m*-Br derivative **25c** and 1.5 for *p*-Br derivative **25d**.

Additionally, there was a report of a promising alkylene amine compound **26b** with a 0.16 µM IC_50_, whereas the enhancement of camptothecin cytotoxicity (ratio of CC_50_ of pure camptothecin to CC_50_ in the presence of **26b**) was 10.47 [57]. It should be noted that its isomer with a shorter spacer linking the aromatic ring to the backbone of usnic acid (compound **26a**) does not sensitize the MCF-7 cell line to the cytotoxic effect of topotecan but has an IC_50_ of 0.19 µM. Compound **26b** was next investigated as a sensitizer for the antitumor activity of topotecan in vivo [58]. A sensitizing effect of compound **26b** (when administered intragastrically) on Lewis lung carcinoma in mice in combination with topotecan was documented. The combination of topotecan with this usnic acid derivative significantly diminished both the volume of the primary tumor and the number of metastases. The absence of acute toxicity of this compound was demonstrated, as well as the importance of the administration method for the manifestation of the sensitizing properties. In addition, a macroscopic examination of the lungs of mice with lung Lewis carcinoma treated with the combination of topotecan and compound **26b** revealed a decrease in metastatic scores by 91%, simultaneous with the inhibition of metastases of up to 98%. Morphological and morphometric analyses of lung sections revealed the elevation of a metastasis growth delay index to 86% at the inhibitor dose of 4 mg/mouse [59].

In a series of terpenyl-substituted usnic acid enamines, compounds capable of inhibiting TDP1 at submicromolar concentrations have also been identified [60]. Among them, there were enamines containing acyclic terpene or bicyclic pinene substituents (compounds **27**, Figure 8) and with IC_50_ values in the 0.23–0.40 μM range. The distancing of the terpene substituent from the usnic acid backbone via the introduction of an amino acid linker (compounds **28**, Figure 8) resulted in a complete loss of the inhibitory activity. Molecular modeling indicated that derivatives with relatively short aliphatic chains fit important binding domains. Intrinsic cytotoxicity of active compounds was tested on two human cell lines: HeLa (cervical carcinoma) and HEK293 (human embryonic kidney). Compounds **27** manifested low cytotoxicity, with CC_50_ ≥ 60 μM toward both cell lines. The compounds enhanced topotecan’s toxicity to cancerous HeLa cells but reduced it toward noncancerous HEK293A cells.

**Figure 9:**
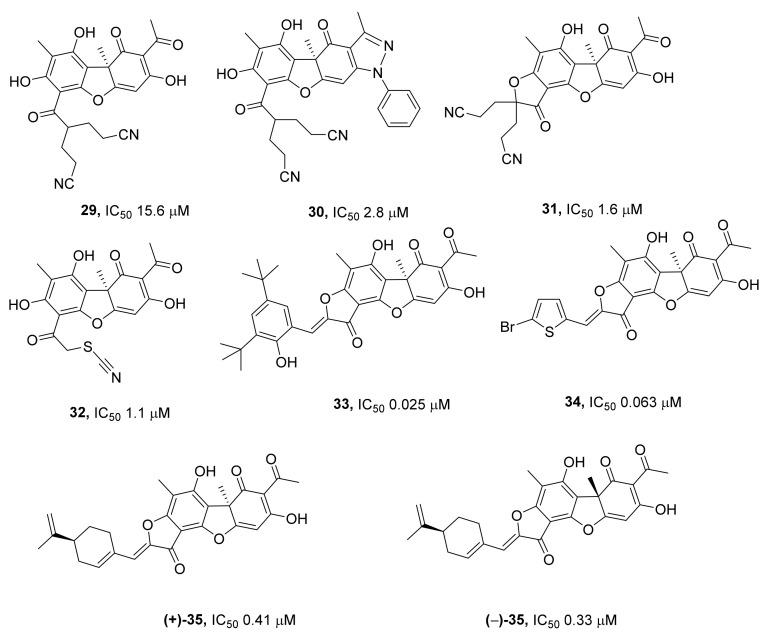
Cyano and furanone derivatives of usnic acid as inhibitors of TDP1.

In ref. [61], researchers studied the inhibitory properties of derivatives of usnic acid modified in another part of the molecule (on ring A) via the introduction of substituents containing a cyano group (nitrile moiety). In an in vitro assay, compounds **29**–**32** (Figure 9) showed effective inhibition of TDP1 with an IC_50_ of 1–3 μM. In this study, compound **30** carrying a pyrazole ring on the C-ring did not have a toxic effect on mammary adenocarcinoma MCF-7 cells (in contrast to other cyano derivatives of usnic acid) at all studied concentrations up to 100 μM inclusively. Those authors attribute the lack of cytotoxicity of this compound to the modification of ring C, which is responsible for the cytotoxicity of usnic acid [56]. As shown in ref. [62], this compound exerts its own antitumor action on Krebs-2 ascitic carcinoma (reduces the concentration of tumor cells) and Lewis lung carcinoma (diminishes both the weight of the primary tumor nodule and the number of lung metastases) in vivo. Compound **30** did not affect the weight of the liver and spleen of the mice, implying the absence of acute toxic effects. It was also noteworthy that the treatment with this compound normalized erythrocyte and leukocyte counts in the blood of the mice; these counts were altered by the tumor in this model [62].

Several series of usnic acid derivatives containing aromatic, heteroaromatic, or terpene substituents attached to the aromatic ring of usnic acid through an annelated furanone moiety are described as effective inhibitors of TDP1 in refs. [63,64]. Excellent inhibition was obtained when aryl and hetaryl derivatives were tested for in vitro TDP1 inhibitory activity by a fluorescent assay, with derivative **33** (Figure 9) showing the lowest IC_50_ value of 25 nM. The observed efficacy was verified in a gel-based assay, which yielded virtually the same results. A synergistic effect of the TDP1 inhibitors with topotecan was tested on two human cell lines, A-549 (human alveolar basal epithelial adenocarcinoma) and HEK293 (human embryonic kidney). Compound **34** (Figure 9) showed low cytotoxicity and an IC_50_ of 63 nM. Among terpenyl derivatives, the most potent compounds had IC_50_ values in the range of 0.33 – 2.7 µM. The inhibitory properties were mainly dependent on the flexibility and length of the terpenoid moiety but not strongly dependent on the configuration of the asymmetric centers. Thus, for a pair of diastereomeric derivatives of (+)- and (−)-usnic acids containing perillyl substituent (+)-**35** or (−)-**35** (Figure 9), IC_50_ values were 0.41 and 0.33 μM, respectively. The synthesized derivatives had low toxicity to human cell lines in an MTT assay.

**Figure 10:**
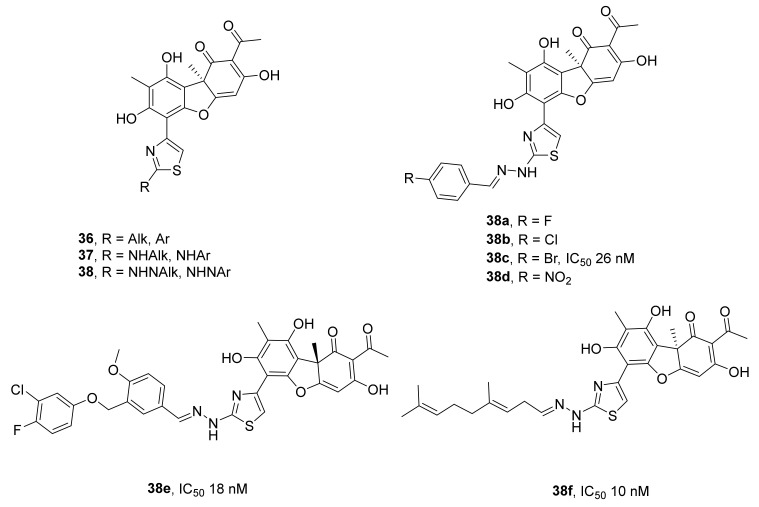
Thiazole derivatives of usnic acid as inhibitors of TDP1.

Another series of derivatives of usnic acid modified by the introduction of various substituents into ring A through a thiazole linker (compounds **36–38**, Figure 10) was described in ref. [65]. It was reported there that in the series of these derivatives, compounds with alkyl, aryl, aminoalkyl, or aminoaryl substituents in the thiazole cycle were good inhibitors (IC_50_ values ~1 mM). A significantly higher activity, by 1–2 orders of magnitude, was shown by compounds **38**, which contain a thiazole ring with an arylhydrazone substituent. Of all the tested compounds, four hydrazinothiazole derivatives that are fluoro-, chloro-, bromo-, or nitro-substituted on the aromatic ring of the hydrazone moiety (compounds **38a–d**) inhibited the enzyme in the nanomolar range. The most effective TDP1 inhibitor, **38c** (IC_50_ = 26 nM), was moderately toxic to cell lines MCF-7 and LLTC (CC_50_ = 1.7 for both) and enhanced the effect of topotecan on both cell lines. Furthermore, compound **38c** significantly strengthened antitumor and antimetastatic effects of topotecan on Lewis lung carcinoma in mice [65,66].

Replacing an aromatic substituent with a heteroaromatic substituent in hydrazone derivatives [67] overall indicates that the activity against TDP1 indeed depends on the structure of the substituent, and the IC_50_ varies from 44 nM to >1.5 μM. An increase in the length of the substituent in the hydrazone moiety made it possible to obtain compounds with the lowest IC_50_ values; for example, for compound (−)-**38e** (Figure 10), the IC_50_ was 18 nM.

A dependence on the stereochemistry of the molecule was observed, e.g., compound **38′**s second isomer obtained from (+)-usnic acid inhibited TDP1 noticeably worse (for (+)-**38,** the IC_50_ was 77 nM). The authors hypothesized that the enlargement of the molecule leads to a tighter fit of the ligand to the active site and consequently to a greater influence of the structural features on the inhibitory activity.

This supposition was proven in a subsequent study, where a series of hydrazinothiazole derivatives with different monoterpenoid fragments was synthesized, and their inhibitory activity toward TDP1 was measured [68]. The inhibitory properties of the new compounds were shown to depend mainly on the structure of the terpene moieties. The most potent compound (**38f**, Figure 10) was synthesized from citral (acyclic monoterpenoid) and has an IC_50_ of 10 nM. Some synthesized derivatives showed low toxicity to HeLa cells and increased the effect of TOP1 inhibitor topotecan in vitro three- to sevenfold.

**Figure 11:**
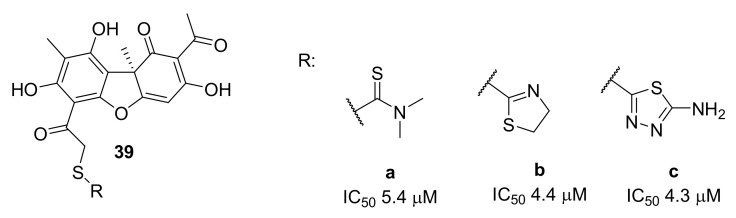
Usnic acid thioether derivatives as TDP1 inhibitors.

Compounds capable of acting as double and even triple inhibitors of repair enzymes have been identified in a series of thioester derivatives of usnic acid [69]. Along with TDP1 inhibitory ability, the inhibitory properties of several compounds toward TDP2 and PARP1 were detected. Usnic acid derivatives **39** (Figure 11) efficiently suppressed TDP1 activity, with IC_50_ values in the 1.4–25.2 μM range. The structure of the heterocyclic substituent connected to the dibenzofuran core through a thioether linker affected the TDP1 inhibitory efficiency of these compounds. A five-membered heterocyclic moiety (compounds **39b–c**) proved to be the most pharmacophoric among the other substituents. An uncompetitive type of inhibition was documented for the four most effective inhibitors of TDP1. Leading compound **39a** showed promising synergy with topotecan toward HeLa cells.

### 3.3. Isoquinolines

**Figure 12:**
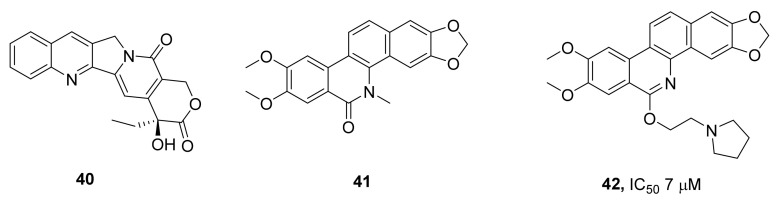
Camptothecin and its analogs that are potential TDP1 inhibitors.

The search for inhibitors of TDP1 among compounds with the isoquinoline backbone has primarily been driven by the fact that the only type of TOP1 inhibitors used in clinical practice are compounds of the isoquinoline alkaloid camptothecin **40** family (Figure 12). Inhibitors of TDP1 have been proposed as an adjunctive therapy for antitumor treatment with inhibitors of TOP1; hence, a combination of the inhibitory properties toward both enzymes within one molecule looked attractive. The authors of ref. [36] have noticed that oxinitidine **41** (Figure 12) may be a suitable scaffold for synthesizing dual inhibitors of TDP1 and TOP1. Three kinds of analogs—benzophenanthridinone, dihydrobenzophenanthridine, and benzophenanthridine derivatives—were synthesized and evaluated for both TOP1 and TDP1 inhibition and cytotoxicity. It was found that compound **42** (Figure 12) caused TDP1 inhibition with an IC_50_ of 7 μM and has a synergistic effect with camptothecin on MCF-7 cells.

**Figure 13:**
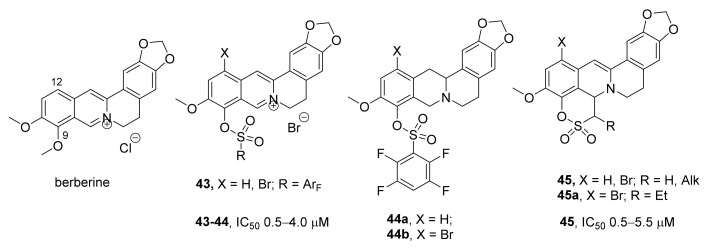
Berberine derivatives that are inhibitors of TDP1.

Berberine (Figure 13) is an alkaloid present in many plants. This compound has antibacterial, antioxidant, hypocholesterolemic, and antitumor effects [70] and inhibits TOP1 [71] but not TDP1. The authors of ref. [72] have demonstrated that in contrast to berberine, its sulfonate derivatives **43** (Figure 13) can inhibit TDP1 in the low micromolar range (0.53–4 μM). It was shown that the addition of a sulfonate group containing a polyfluoroaromatic moiety at position 9 enhanced the potency, whereas most of the derivatives containing an alkyl group or a fluorine-free aryl at the same position were not effective. According to molecular modeling, the bromine atom at position 12 may improve affinity for the enzyme. Indeed, the IC_50_ values of brominated compounds were found to be lower than those of their unsubstituted analogs; an inhibitory activity was detected even in the series of alkylsulfonates where bromine was present at position 12. The observed structure–activity relationship proved to be applicable also to derivatives based on the tetrahydroberberine scaffold. The cytotoxic effect of topotecan on the HeLa cell line was doubled by derivatives **44a** and **44b** (Figure 13); both had low cytotoxicity without topotecan.

In a subsequent work [73], it was shown that in contrast to their noncyclic analogs that did not show activity toward TDP1 [73], compound **45** (Figure 13)—having a sultone ring condensed to *C* and *D* rings of a protoberberine core—suppressed TDP1 activity in the micromolar or submicromolar range (IC_50_ = 0.56–5.5 μM). It was demonstrated in the study [73] that the introduction of a bromine atom increased the inhibitory effect of the compounds. The active inhibitors were mostly nontoxic to the HeLa cancer cell line at concentrations up to 100 μM, and some of these ligands showed synergy with topotecan. Among berberrubine sultones, the most pronounced sensitizing effect was exerted by compound **45a**, which carried a bromine substituent [73].

### 3.4. Terpenoids

#### 3.4.1. Monoterpenoids

In the literature, we failed to find examples of monoterpenes acting as inhibitors of TDP1. On the other hand, there are many examples where a monoterpenoid served as a moiety in such a ligand molecule. When monoterpene groups were introduced into other bulky molecules, it was reported that the monoterpene moieties make a major contribution to the inhibitory activity of the compounds.

**Figure 14:**
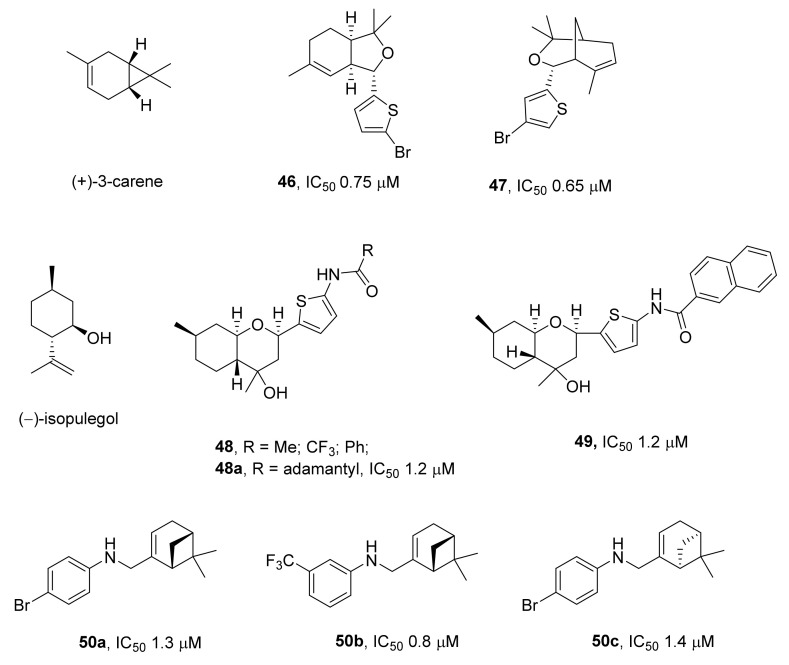
Monoterpenoid derivatives as inhibitors of TDP1.

In ref. [74], derivatives of a bicyclic monoterpenoid called (+)-3-carene, which have hexahydroisobenzofuran and 3-oxabicyclononane backbones, were investigated. Compounds carrying phenyl and alkyl substituents exerted no action against TDP1, whereas the IC_50_ values of substances having heterocyclic substituents varied from 0.65 to 28 μM. The addition of bromine to the heterocycle significantly increased the inhibitory capacity. For example, compound **46** (Figure 14), which contains a bromine atom at the fifth position of the thiophene ring, manifested the best activity in the submicromolar range (IC_50_ = 0.75 μM) among hexahydroisobenzofurans. Among compounds with a 3-oxabicyclo[3.3.1]nonane backbone, 4-bromo-substituted compound **47** was the most effective (IC_50_ = 0.65 μM) [74]. The toxicity of all the compounds to wild-type cells and TDP1-deficient cells (a *TDP1* knockout cell line: HEK293FT TDP1^−/−^) was undetectable or negligible in the concentration range 0.08–100 μM [74]. It was noted that an enhancement of topotecan action by the presence of a TDP1 inhibitor was observed only for wild-type cells and was absent with mutant cells. This result confirms that the synergistic effect of topotecan with these inhibitors was due to the suppression of TDP1 activity by the selected inhibitors. It is also likely that TDP1 is the main target for this type of inhibitor because there was no additional increase in toxicity to mutant cells.

Octahydro-2H-chromenes were obtained from a monoterpenoid called (−)-isopulegol [75]. A possible inhibitory activity of this type of compound was hypothesized in a computer simulation of an interaction of the TDP1 active center with various derivatives of octahydrochromene. Compounds **48** (Figure 14) were found to inhibit TDP1 with an IC_50_ of 1.2 to 15 μM. The most effective inhibitor was compound **48a**, which contained a bulky adamantane substituent. In silico calculations revealed that amide derivatives of thiophenyloctahydrochromene, having bulky lipophilic substituents in the amide moiety, should have the strongest affinity for TDP1. To verify the results of the calculations, respective naphthyl derivatives **49** were synthesized (Figure 14). It was shown that they exerted an inhibitory action on TDP1 in a micromolar concentration range (IC_50_ = 1.24–5.8 μM) [76].

Secondary aromatic amines containing myrtenyl, nopyl, or perillyl moieties are active in terms of TDP1 inhibition in a low micromolar or even submicromolar range of concentrations [77]. In this study, the inhibitory activity was significantly affected by both the nature of the monoterpene moiety and the length of the linker between the phenyl and monoterpene moieties as well as by the nature and position of the substituents in the phenyl moiety. The lowest IC_50_ values were shown by compounds **50a–c** (Figure 14), which contained myrtenyl substituents.

**Figure 15:**
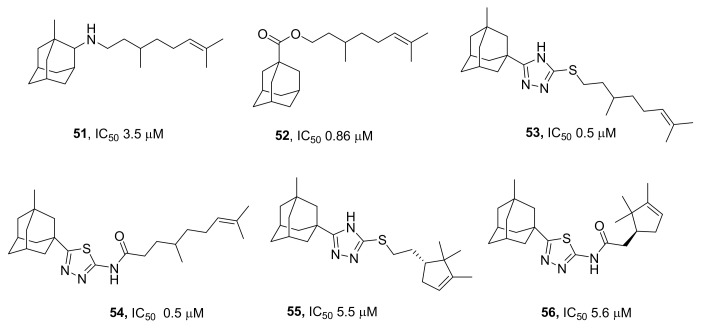
Terpenyl adamantane compounds that are inhibitors of TDP1.

Many research projects have addressed the inhibitory activity of monoterpenoids linked to an adamantane or diazaadamantane moiety via spacers of various structures [78,79,80,81,82]. The structure of terpenoid substituents has been varied, and not only acyclic (geranyl or citronellyl) but also monocyclic (peryl or campholene) or bicyclic (pinane or bornane) backbones have been used. Almost all the above data indicated that a long flexible acyclic terpene substituent at position 2 was required for the inhibition of TDP1 activity. Among amino- and imino-derivatives of adamantane (IC_50_ = 3.5 to 34 μM) in a series of terpene substituents, the strongest effect was shown by derivative **51** (Figure 15), which contained a hydroxycitronellyl substituent [79,83]. Compound **51** potentiated the effect of topotecan on human colon cancer HCT-116 cells by more than fivefold [79].

Furthermore, acyclic monoterpene substituents correlated with greater inhibitory activity in a series of terpenyl-substituted diazaadamantane derivatives, and their IC_50_ varied from 15 µM (acyclic terpene substituents) to 100 µM (bicyclic terpene substituents) [78].

Derivatives containing an acyclic monoterpene substituent attached to adamantane through an ester group, amide group, or thioamide group have proven to be better inhibitors than derivatives containing a monocyclic or bicyclic monoterpene substituent [80,81]. At 0.86 μM, the most active compound **52** (Figure 15) decreased the activity of the TDP1 enzyme by 50%.

A similar trend was observed among terpenyl adamantanes in which pharmacophores were linked via a heterocyclic spacer, i.e., via a 1,2,4-triazole or 1,3,4-thiadiazole linker [82,84]. Citronellyl-substituted derivatives **53** and **54** (Figure 15) inhibited TDP1 with an IC_50_ of 0.5 µM, while the corresponding campholene derivatives inhibited it with an IC_50_ of 5.5 µM, and bornyl derivatives with an IC_50_ > 30 µM. In relation to topotecan, terpenyl adamantanes having a triazole linker were found to have a sensitizing effect on cervical cancer HeLa cells and colon adenocarcinoma HCT-116 cells [82].

Campholenic derivatives **55** and **56** inhibited TDP1 at a low micromolar range: IC_50_ ≈ 5–6 µM [84]. These compounds manifested clear synergy with topotecan regardless of their ability to inhibit TDP1. The authors attributed this finding to the probable existence of other pathways mediating the increase in sensitivity to topotecan [84].

Terpenylcoumarins were discussed above (Section 3.2, Figure 7). It should be briefly mentioned that, just as in terpenyl adamantanes, in a series of terpene-substituted 7-hydroxycoumarins [52], derivatives with acyclic substituents were the most effective (compound **21**, Figure 7, IC_50_ = 0.62 μM). By contrast, in a series of terpenylcoumarins carrying an aryl substituent, compounds with a bicyclic terpene substituent (compound **20**, Figure 7) had comparable effectiveness (IC_50_ = 0.68 μM) [53].

There are similar trends among terpenic derivatives of usnic acid (compounds **27**, Figure 8) [60]. Enamines containing acyclic terpene substituents or bicyclic pinene substituents have turned out to be the most effective, with IC_50_ values in the 0.23–0.40 μM range. Acyclic monoterpene substituents enhance the inhibitory activity in the hydrazonothiazole series (compound **38f**, Figure 10) as compared to their analogs with mono- and bicyclic terpene substituents. Moreover, only in a series of furanone derivatives, the leading compound (**35**) contained a monocyclic peryllic substituent (Figure 9).

#### 3.4.2. Diterpenoids

Among the diterpene compounds, only derivatives of dehydroabiethylamine (a component of coniferous resins) have been tested for TDP1 inhibitory activity. It is known that dehydroabiethylamine hydrochloride is highly toxic to some tumor cell lines and can kill melanoma cells by raising the level of apoptosis and reducing the proliferation rate [85]; the latter phenomena possibly contribute to its potential antitumor activity.

**Figure 16:**
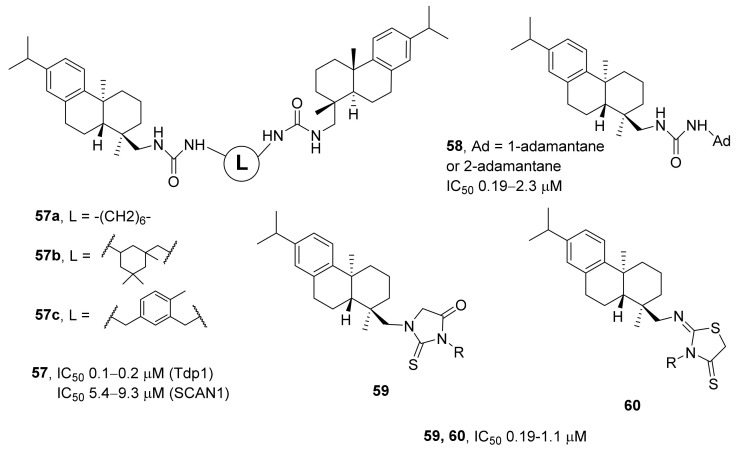
Dehydroabiethylamine derivatives that are inhibitors of TDP1.

In ref. [86], the researchers obtained dehydroabiethylamine derivatives of urea, thiourea, and bis-urea. A study on their activity against TDP1 revealed IC_50_ values of 0.1 to 3.7 μM. The best inhibitory effect on both wild-type TDP1 and the H493R mutant (SCAN1) was exerted by derivatives of dehydroabiethylamine belonging to the bis-urea class (compounds **57**, Figure 16). the IC_50_ values for bis-ureas varied in the range from 0.1 to 0.2 μM for TDP1 and from 5.4 to 9.3 μM for SCAN1. At concentrations up to 100 μM, these compounds were not toxic to various immortalized cell lines. The ability of these compounds to enhance the effect of temozolomide (an alkylating agent used in the chemotherapy of glioblastomas and metastatic melanomas) was studied as well. The combination of temozolomide with an inhibitor of TDP1 (compounds **57a,b**, Figure 16) lowered the viability of human glioblastoma cell lines U87MG and SNB19 by as much as 40% as compared with temozolomide alone.

Synthesized abiethylamine derivatives with an adamantyl urea substituent inhibit TDP1 at micromolar concentrations (IC_50_ = 0.19–2.3 μM) [87]. Adamantane derivatives of resin acids **58** (Figure 16) showed moderate toxicity to T98G cells (glioblastoma). Cell viability in the presence of 2.5 or 5 μM inhibitors was in the range of 90–100%, and the cytotoxicity increased only at a compound concentration of 25–50 μM but did not exceed 60% (percentage of dead cells). At a concentration of 5 μM, the compounds were also shown to have an additive effect with 2 mM temozolomide on T98G cells.

In a series of dehydroabiethylamine derivatives carrying a 2-iminothiazolidine-4-thione or 2-thioxoimidazolidine-4-thione moiety (compounds **59** and **60**, Figure 16), the IC_50_ values were in the same concentration range as compared to adamantyl derivatives (0.19 to 1.1 μM) [88]. For heterocyclic derivatives, a pattern was noted: enlargement of the substituent lowered the IC_50_ [88].

#### 3.4.3. Sesqui- and Triterpenoids

Several sources have indicated that triterpene compounds hold promise as inhibitors of TDP1. For instance, it was noted in ref. [89] that a triterpene fraction of an extract from *Antrodia cinnamomea* strongly inhibited the cellular expression of TOP1 and TDP1 both at transcriptional and protein levels. To clarify the mechanism by which this triterpenic fraction reduces tumor cell viability, the investigators measured TDP1 and TOP1 amounts in HeLa cells by an enzyme-linked immunosorbent assay. After 24 h in cells treated with ACTs-E at concentrations of 100, 200, or 400 μg/mL, both TOP1 and TDP1 levels were significantly (*p* < 0.05 or *p* < 0.01) lower than those in the control group.

**Figure 17:**
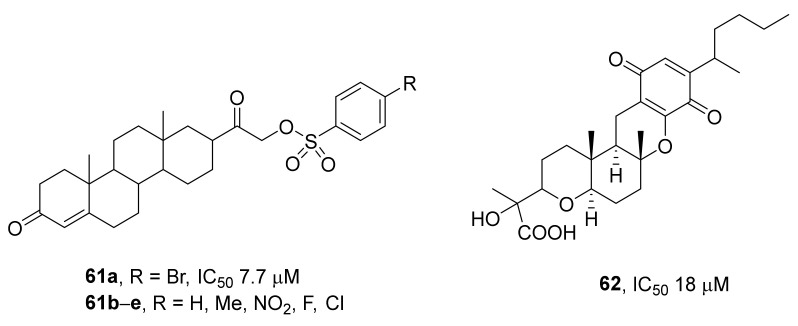
TDP1 inhibitors based on steroid derivatives.

A screening revealed a new potential inhibitor of TDP1: C21-substituted progesterone derivative NSC 88915 (compound **61a**, Figure 17) [90]. Replacing the bromine atom in compound NSC 88915 with various substituents (compounds **61b–e**, R = H, CH_3_, NO_2_, F, or Cl) resulted in minimal changes in the inhibition of TDP1 (~3-fold change in IC_50_ values). It was theorized that these compounds were competitive inhibitors that mimic an oligonucleotide–peptide substrate of TDP1.

A recently found natural compound, JBIR-21 (**62**), isolated from an anamorphic fungus [91], was reported to inhibit TDP1 with an IC_50_ of 18 μM, to be toxic to cancer cell lines (HeLa, NCI-H2052, HT-29, and Namalva; IC_50_ values of 9.2, 13, 4.0, and 3.5 μM, respectively), and to exert antitumor activity against HT-29 cells in a mouse xenograft model.

**Figure 18:**
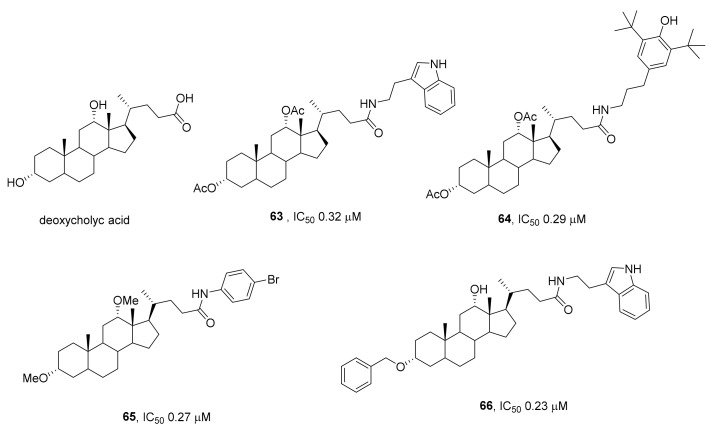
TDP1 inhibitors based on deoxycholic acid amides.

Potential inhibitors of TDP1 based on a skeleton of bile acids have been recognized as promising according to virtual screening involving an assessment of binding of ligands (from the InterBioScreen library of natural compounds) to the active site of the enzyme [92]. In vitro screening showed that derivatives of three bile acids (ursodeoxycholic, chenodeoxycholic, and deoxycholic) had comparable inhibitory activity against TDP1 (IC_50_ ≈ 2.6 μM). The authors of that work chose the most accessible bile acid (deoxycholic) for the synthesis of new derivatives. As a result, derivatives were obtained that had a higher inhibitory effect on TDP1 than native deoxycholic acid. Amides containing moieties of tryptamine, para-bromoaniline, aminoadamantane, or butylphenol showed inhibitory properties with IC_50_ values in the range of 0.29–0.47 μM. The most effective deoxycholic acid amide were compounds **63** and **64** (Figure 18, IC_50_ = 0.32 μM and 0.25 μM, respectively). Assays of cytotoxicity of diacetoxy derivatives of deoxycholic acid on human breast adenocarcinoma (MCF-7) and human colon carcinoma (HCT-116) cells indicated that the toxicity of the compounds was undetectable or negligible at concentrations up to 100 μM [92].

To evaluate the effect of functional groups of the steroid backbone on the TDP1 activity, researchers have synthesized and tested derivatives of deoxycholic acid *para*-bromoanilides containing acetoxy, hydroxy, or carbonyl groups as well as ester groups (methoxy, ethoxy, or propyloxy) on the backbone of deoxycholic acid [93]. All of them were able to inhibit TDP1 with IC_50_ values in the range of 0.27–1.54 μM. A 3,12-dimethoxy compound containing a *p*-bromoanilide substituent caused the strongest inhibition (IC_50_ = 0.27 μM) (compound **65**; Figure 18) [93]. An assay of the antiproliferative activity of deoxycholic acid *para*-bromoanilides toward tumor cell lines revealed that the compounds are nontoxic to A549 cells (lung adenocarcinoma), whereas the toxicity to HCT-116 cells (human colon carcinoma) and HepG2 cells (hepatocellular carcinoma) directly depended on the functional groups attached to the backbone of deoxycholic acid. Compounds with a free hydroxyl group turned out to be more toxic than derivatives with a hydroxyl group transformed into an acetoxy, oxo, or methoxy moiety.

To investigate the effect of bulky substituents attached to hydroxyl groups in bile acids’ framework on the inhibitory activity of relevant compounds, substances have been synthesized containing a benzyl, ether, or aromatic moiety (p-bromobenzene, indole, or 2,6-bis-tert-butylphenol) attached to the steroid through various linkers [94]. All the compounds inhibited TDP1 in a submicromolar concentration range (IC_50_ = 0.23–1.2 μM) and had low intrinsic toxicity to HEK293A (human embryonic kidney) cells and HeLa (cervical carcinoma) cells. Tryptamide derivative **66** (Figure 18) (containing a benzyloxy substituent at the C-3 position and an unsubstituted hydroxyl group at the C-12 position) and a *para*-bromoanilide derivative (containing similar substituents at the C-3 and C-12 positions on the bile acid backbone) also enhanced (twofold) the cytotoxic effect of topotecan on HeLa cells.

## 4. Conclusions

Inhibitors of TDP1 have been studied since the 2000s. To date, many compounds have been synthesized and investigated as inhibitors of TDP1. Our earlier reviews have covered mainly synthetic inhibitors [95] and different approaches to the development of TDP1 inhibitors [96]. This review shows considerable recent progress in the design of TDP1 inhibitors based on natural compounds. Our analysis of the literature suggests that natural compounds are a promising platform for the development of effective inhibitors of this enzyme.

Various classes of compounds have been researched as inhibitors of TDP1, such as, for example, mono-, di-, and triterpenoids; alkaloids; glycosides; and phenolic compounds. Most of the studied natural compounds inhibit TDP1 at micromolar or higher concentrations. Nonetheless, chemical modifications of natural compounds allow obtaining more effective inhibitors, which work at submicromolar and even nanomolar concentrations.

In the search for effective inhibitors among natural compounds and their derivatives, two main approaches have been used: screening via molecular modeling (in silico) and screening for inhibitory activity in lab-made libraries (in vitro). Although their structures are very different, most compounds inhibit TDP1 in the same concentration range (10^−5^–10^−7^ M). Furthermore, compounds with in silico-predicted inhibitory properties have not shown a stronger inhibitory effect in in vitro assays as compared to compounds identified by experimental screening.

An analysis of the structures of the most potent inhibitors allows distinguishing some determinants of stronger affinity for the enzyme. The most potent inhibitors as a rule contain a fairly large core of the molecule in the form of several fused rings: aromatic (coumarins, berberines, and dibenzofurans), saturated (triterpenic acids and adamantanes), or a combination of the two (diterpenes). Sometimes, a fairly large size and length of this bulky moiety (usually five rings or more) are enough for inhibition of the enzyme at micromolar concentrations (compounds **45** and **62**). Most often, however, to achieve effective inhibition, a “tail” of the molecule has been built up in the form of a small unsaturated (terpenoids), aromatic, or heteroaromatic moiety linked to the core of the molecule through a flexible spacer. Often, it is the structure of this flexible part of the inhibitor molecule that has proven to be crucial for reducing the inhibitory concentration by an order of magnitude. An analysis of structures of compounds in libraries of inhibitors from various classes also helps to identify the most common small structural components: pinene, *para*-bromophenyl, 4- or 5-bromothiophene, and 3,5-di-tert-butyl-4-hydroxyphenyl substituents. Either aliphatic (C-2 or C-3) or heterocyclic moieties have been employed as spacers.

An important stage in the verification of the desired activity of such compounds is the testing of a combination of such an inhibitor with an anticancer chemotherapeutic drug. Synergy or a sensitizing effect of such a combination therapy has not been detected for all compounds in experiments in vitro; most of the ineffective sensitizers (compounds **48–50**, Chepanova, unpublished data) are relatively small molecules, and it is possible that these substances have multiple targets—in particular, undesirable preferred targets—in the cell (PAINS).

Nonetheless, for most compounds that have been studied, the inhibitory activity observed in screening tests on the purified enzyme has been confirmed in experiments on cultured cells, as evidenced by synergy between the TDP1 inhibitor and an antitumor drug. Be that as it may, the most important step is to confirm the effectiveness of the inhibitor in experiments in vivo. To date, only a few such reports have been published. Synergistic effects of derivatives of nucleosides (compound **10**), usnic acid (compounds **26b** and **38c**), or coumarins (compound **21**) with an antitumor drug have been confirmed in animal experiments.

Among the derivatives of natural compounds, substances with double or triple inhibitory activity (toward TDP1 and other enzymes of DNA metabolism) have also been identified. Such properties in a single low-molecular-weight compound may offer major advantages over drugs acting on a single target. The suppression of several targets at once by a single inhibitor may help to reduce the number and doses of anticancer drugs prescribed within a treatment course. On the other hand, such compounds often have high intrinsic cytotoxicity due to the effects on several important enzymes at once. In the future, this property may complicate finding an optimal dose and their use in combination with other anticancer drugs. In addition, such drugs are not designed to address individual differences among patients in terms of the levels of expression and/or activity of target enzymes; this shortcoming also potentially complicates the choice of the dose.

In fact, the overall effect of a TDP1 inhibitor in vivo is composed of several factors: enzyme affinity, specificity, toxicity, and bioavailability. Evaluating the compounds presented in this review, it can be noted that in the presence of a good affinity for the enzyme, the weak points of nucleosides are the nonspecificity of action, while terpenic compounds and berberines have poor bioavailability. Among phenolic compounds characterized by good bioavailability, coumarins are multitarget, that is, nonspecific, and usnic acid derivatives, although they have excellent affinity for the enzyme, are often quite toxic. Apparently, further efforts in the development of effective TDP1 inhibitors based on natural compounds should be made, focusing on these aspects.

Nevertheless, the fact that several lead compounds have manifested promising effects in in vitro screening, in-cell experiments, and in vivo experiments shows the promise of TDP1 inhibitors as components of anticancer therapy. Therefore, the search for new inhibitors of TDP1 remains a relevant research aim.

## Data Availability

Data sharing is not applicable.

## Abbreviations

PLD	phospholipases D family enzymes
SCAN	spinocerebellar ataxia syndrome with axonal neuropathy
TDP	tyrosyl-DNA phosphodiesterase
TOP1	topoisomerase 1
